# Recurring transient brain-wide co-activation patterns from EEG spatially resembling time-averaged resting-state networks

**DOI:** 10.1162/IMAG.a.1202

**Published:** 2026-04-10

**Authors:** KC K. Nkurumeh, Han Yuan, Lei Ding

**Affiliations:** Stephenson School of Biomedical Engineering, University of Oklahoma, OK, Norman, United States; Institute for Biomedical Engineering, Science, and Technology, University of Oklahoma, OK, Norman, United States

**Keywords:** co-activation pattern, electroencephalography, resting-state network, independent component analysis, temporal dynamics, functional connectivity

## Abstract

It has long been established that human brains remain functionally active at rest, as demonstrated with the discovery of resting-state networks (RSNs) underlying spontaneous neural activity. Recent studies suggest that classical RSNs estimated from functional magnetic resonance imaging (fMRI) data using time-domain functional connectivity measures might be driven by recurring point-process events. Due to the slow hemodynamic response, fMRI cannot reveal such point-processes at the timescale of neuronal events while electroencephalography (EEG) holds the promise due to its millisecond temporal resolution and successful reconstruction of fMRI-like RSNs. The present study reported a set of recurring transient (<100 ms) cortical co-activation patterns (CAPs) derived from resting-state EEG using a clustering algorithm with spatial-domain measures (i.e., k-means). Our results indicate that this set of CAPs exhibit strong spatial correspondence with known RSNs, not only those derived from the same EEG data using time-domain measures (i.e., independence), but also those from fMRI literature, covering visual, auditory, motor, limbic, high-order, and default mode networks. CAPs exhibit the properties of hemispheric symmetry, spatially separatable sub-systems, and intersubject variability gradient across functional systems, which have all been observed in classical RSNs. These findings suggest that classical RSNs might be driven by recurring transient neuronal activations captured in CAPs. More importantly, CAPs can reveal the fast dynamics of such brain-wide networked neuronal activations (e.g., different CAPs exhibit significantly different occurrences and lifetimes) and benefit from their intersubject reproducibility, thus underscoring their potential to advance our understanding on neuronal mechanisms of spontaneous large-scale brain activation phenomena.

## 1 Introduction

Human brains are organized into multi-layer networks, and such hierarchical structures serve as neural substrates for distinct brain functions (Biswal et al., 1995; Fox & Raichle, 2007), especially for high-level functions such as cognition (Bressler & Menon, 2010). These networks can be modulated by tasks (Betti et al., 2013; Krienen et al., 2014) while their intrinsic natures are present even without explicit tasks (Cole et al., 2014). Such intrinsic phenomena have been widely captured in neuroimaging data, for example, functional magnetic resonance imaging (fMRI), electroencephalography (EEG), magnetoencephalography (MEG), and functional near-infrared spectroscopy (fNIRS). Their spatial constructions are obtained via calculating functional connectivity (FC) measures among signals recorded between different locations, known as resting-state networks (RSNs) (Jafri et al., 2008). The properties of these RSNs have shown significant behavioral correlates (Rosenberg et al., 2016) and have been associated with clinical abnormalities in major neuropsychiatric disorders (Fox & Greicius, 2010; Friston, 2011).

While RSNs were first reconstructed in a static, time-averaged fashion from multi-minute recordings of fMRI blood-oxygen-level-dependent (BOLD) data using data-driven FC measures, for example, pairwise temporal correlation (Biswal et al., 1995; Fox et al., 2005) or spatial independence via independent component analysis (ICA) (Beckmann et al., 2005; Fox & Raichle, 2007), later studies have demonstrated that RSNs show dynamics over the timescale of minutes in fMRI data (Chang & Glover, 2010; Hutchison et al., 2013; Iraji et al., 2019a, 2019b; Kiviniemi et al., 2011). Some recent studies further indicate that fMRI-based network dynamics at much shorter temporal scales (i.e., ~22.5 seconds) can be used in tracking cognition while performing multiple tasks (Gonzalez-Castillo et al., 2015), which suggest potential sub-minute FC dynamics in RSNs. In EEG/MEG scenarios, these time-domain FC measures, for example, correlation (De Pasquale et al., 2010) and independence (Li et al., 2018), and their corresponding sliding-window versions (Shou et al., 2020), have been successfully utilized to reveal both static and dynamic RSNs. Due to their millisecond (ms) temporal resolutions, EEG/MEG signals hold the potential in revealing RSN dynamics at the timescale of neuronal events (i.e., tens to hundreds of milliseconds). The adoptions of novel measures, for example, spatial-domain measures (as opposed to time-domain FC measures) (Liu & Duyn, 2013), advanced methods, for example, Hidden Markov Methods (HMM) (Baker et al., 2014; Vidaurre et al., 2017), and other ad-hoc algorithms (Gu et al., 2021; Matsui et al., 2016) have led to the identifications of several new large-scale patterns in both fMRI and EEG/MEG at the temporal resolution of individual timeframes (only limited by sampling rates of corresponding neuroimaging modalities). While most of these large-scale patterns, for example, propagational waves (Matsui et al., 2016), co-activation patterns (CAPs) (Liu & Duyn, 2013), and HMM brain states (Baker et al., 2014), are typically not named as RSNs as they are not constructed using time-domain FC measures, they share similar characteristic brain-wide spatial distributions as RSNs but usually are of transient and recurring nature. In particular, such EEG/MEG-based large-scale patterns exhibit their lifetimes at the timescale of neuronal events, that is, tens to hundreds of milliseconds (Ding et al., 2022; Vidaurre et al., 2018).

Such a collection of large-scale patterns spanning the timescales from tens of milliseconds to several minutes, observable in various signals of fMRI, MEG, and EEG, provides unique opportunities to understanding neuronal mechanisms of classical time-averaged RSNs obtained using time-domain FC measures (Preti et al., 2017). Firstly, the spatial similarity between EEG- and fMRI-derived RSNs suggests a possible shared neuronal basis among them (Yuan et al., 2016). Secondly, recurring fMRI CAPs spatially resemble fMRI RSNs, which may contribute to the emergence of RSNs observed over longer time windows (Liu et al., 2013). Thirdly, the detection of both CAPs and RSNs in human and animal models points to their evolutionary relevance as functional motifs (Belloy et al., 2018). Lastly, while both EEG RSNs and EEG CAPs have been reported and some spatial similarities have been discussed (Ding et al., 2022), current evidence suggests more discrepancies (Shou et al., 2022) in their spatial correspondence than those seen between fMRI RSNs and fMRI CAPs. It is, therefore, of interest to study whether there is another set of EEG CAPs that exhibit more similarities to EEG RSNs, which can fill the missing link between large-scale transient patterns and large-scale time-averaged patterns in EEG signals as observed in fMRI signals.

In the present study, we reported a set of EEG-derived CAPs that resemble time-averaged RSNs identified using a classical temporal ICA approach. These CAPs were constructed using k-means clustering with correlation as the distance measure from cortical envelope data that were inversely reconstructed from surface EEG. The outcome from the present study reveals several important phenomena. Firstly, RSN-like patterns (i.e., CAPs) could be identified at the timescale of typical neuronal events (<100 ms), which is orders shorter than time windows typically used for calculating time-averaged RSNs. Secondly, these CAPs occur repeatedly within participants and are reproducible across participants. Thirdly, several canonical distinctive features observed in RSNs are also observed in CAPs, including hemisphere symmetry, intersubject variability gradient from low-level perceptual systems to high-level functional systems, and existence of both default mode network (DMN) and task positive networks (TPNs). Finally, CAP spatial patterns remain stable across a range of values for a key method parameter, demonstrating their robustness to methodological factors. These observations support the hypotheses that there are large-scale transient patterns matched to large-scale time-averaged patterns in resting human brains.

## 2 Materials and Methods

### 2.1 Data acquisition and preprocessing

The study was approved by IRB at the University of Oklahoma Health Sciences Center (OUHSC) and written informed consent was obtained from all participants before data acquisition. EEG and MRI data from 34 healthy participants (age = 24 ± 5 years, 9 females, absence of known brain diseases or neurological complications) were acquired. Participants were instructed to remain seated upright with eyes closed during EEG recordings. Resting-state EEG data were recorded for 10 minutes, at the sampling rate of 1000 Hz, using a 128-channel Amps 300 amplifier (Electrical Geodesics Inc., OR, USA) in each participant. Positions of EEG sensors along with three landmarks (nasion, left and right pre-auricular points) were digitized by the Polhemus Patriot system. Structural MRI (sMRI) covering full head was collected for each participant on a GE MR750 scanner at the OUHSC MRI facility, using the GE BRAVO sequence: FOV = 240 mm, axial slices per slab = 180, slice thickness = 1 mm, image matrix = 256 × 256, TR = 8.45 ms, TE = 3.24 ms.

All EEG preprocessing was performed using the EEGLAB toolbox (Delorme & Makeig, 2004) separately for individuals. A notch filter of 58–62 Hz and a bandpass filter of 0.5–100 Hz were applied. Noisy channels were automatically identified using the FASTER plugin (Nolan et al., 2010) for EEGLAB and interpolated using data from their neighboring channels. ICA was then applied to identify and remove artifactual components related to cardiac, ocular, or muscular activity by visual inspection of ICs’ topographies, time courses, and spectral properties (Chaumon et al., 2015). Finally, EEG data were downsampled to 250 Hz and referenced to the common average. Note that no time segments of EEG data were removed in order to preserve continuity of data.

### 2.2 Reconstructing cortical source tomography

Cortical source imaging (Li et al., 2018) was used to construct current source distributions over the cortical surface from scalp EEG. Firstly, a boundary element model was generated for each participant. Freesurfer (Fischl, 2012) was used to segment and extract the surface boundaries of the scalp, skull, and brain and the surface boundary between white and gray matters from participant’s sMRI data to create individualized volume conduction models and cortical current density (CCD) source models, respectively. Next, each surface of the volume conduction model was tessellated into 10,242 nodes and 20,480 triangular elements, while the CCD surface was tessellated into 20,484 nodes and 40,960 triangular elements. The nodes along the medial wall comprising the corpus callosum, basal forebrain, and hippocampus were excluded, therefore, reducing the total number of source nodes to 18,715. Each node of the CCD surface was assigned a dipole vector to represent local neuronal currents with its direction defined as the normalized sum of the normal vectors of all triangles sharing the node. The electrical conductivities of the scalp, skull, and brain of the volume conduction model were assigned as 0.33/Ωm, 0.0132/Ωm, and 0.33/Ωm, respectively (Lai et al., 2005). The locations of EEG sensors were registered to the scalp surface by aligning three landmarks from both EEG and sMRI recordings.

Based on the volume conduction and CCD models, the boundary element method (Hamalainen & Sarvas, 1989) was used to build the forward relationship: Φ(t)=L⋅S(t), in which L is an 128 × 18715 lead field matrix representing the contribution of each cortical unity dipole on EEG signals recorded at all sensors and Φ(t) and S(t) are the EEG recordings and dipole source amplitude as functions of time. Based on the forward relationship, the minimum-norm estimate was used to calculate cortical source amplitudes: $\text{S}\left(\text{t}\right)={\text{L}}^{\text{T}}\cdot {\left(\text{L}\cdot {\text{L}}^{\text{T}}+\lambda \left(\text{t}\right)\cdot \text{I}\right)}^{-1}\cdot \Phi \left(\text{t}\right)$ (Hämäläinen & Ilmoniemi, 1994), in which I is the identity matrix, and λ is the regularization parameter for each time instance selected via the generalized cross-validation method (Golub et al., 1979). To control the quality of cortical source estimates, values of λ outside of 3 standard deviations of λ values from all time instances were considered as outliers and interpolated with neighboring values. Based on the adjusted λ values, cortical current source tomography was constructed for individual participants as functions of time.

### 2.3 Estimating time-averaged RSNs with group-level ICA and participant-level statistical regressions

To estimate time-averaged RSNs, group-level time–frequency ICA was performed, a special case of temporal ICA that utilizes spectral time series derived using the Short-Time Fourier transform (STFT) (Bingham & Hyvärinen, 2000; Shou et al., 2012), on the alpha band (8–12 Hz) of the preprocessed EEG data. Alpha band data were chosen because it is the dominant rhythmic neural oscillation in resting human brains (Kirschfeld, 2005). To perform the group-level time–frequency ICA, each participant’s data were converted to z-scores channel-wise to remove inter-participant variabilities. STFT was used to calculate the complex spectral time series representation of time-domain individual EEG data (0–100 Hz, 1 Hz steps, 1 second window, no overlap). Complex spectral EEG time series in the range of 8–12 Hz were averaged across frequency bins to capture the entirety of alpha band, then temporally concatenated across all individuals, and subject to the time–frequency ICA. Various numbers of ICs (i.e., 25, 36, 48, and 64) were investigated, with the maximal number investigated limited to 64 as more ICs (>64) tended to generate focal and regional patterns (Smith et al., 2009). Results from 48 ICs were selected for the subsequent analysis to minimize model complexity while retaining sufficient number of valuable components. To obtain IC time courses, original alpha-band EEG data were projected using the demixing matrix obtained in ICA. ICs showing neural activation characteristics in both their spatial and spectral patterns comparable with neuronal ICs reported in the literature (Brookes et al., 2011; Shou et al., 2012, 2020; Yuan et al., 2016) were selected as the scalp-level representations of time-averaged RSNs, which led to the selection of 29 neuronal ICs out of total 48 ICs (see Supplementary Fig. S1).

Cortical representations of time-averaged RSNs were estimated via the statistical regression analysis (Ding et al., 2022) between the time courses of ICs and cortical dipole sources (see Section 2.2) at individual participants. Firstly, cortical dipole time courses were bandpass filtered to the alpha band (i.e., 8–12 Hz). Secondly, instantaneous amplitudes of the time courses of both neuronal ICs and dipoles were estimated using the Hilbert transform (Baker et al., 2014; Coquelet et al., 2022), and converted to z-scores timewise. Thirdly, a regression was performed for each cortical dipole with the dipole amplitude time course as the response data and the amplitude time courses of all 48 ICs (29 neuronal and 19 non-neuronal ICs) as the regressors. Regression in this manner resulted in participant-level spatial maps of beta-values for each regressor (i.e., each IC). The group-level cortical RSNs were calculated as the average of corresponding participant-level cortical tomography from individual ICs.

### 2.4 Estimation of CAPs: Clustering

To estimate CAPs, cortical dipole time courses were subject to the k-means clustering algorithm at the resolution of individual timeframes. Instantaneous amplitudes of cortical dipole time courses from individual participants that were bandpass filtered to the alpha band (Section 2.3) were used. A dimension reduction from 18,715 cortical sources to 200 regions of interest (ROIs) was performed using a well-established Schaefer atlas for human cortex (Schaefer et al., 2018). The ROI-based cortical source amplitude data were calculated as the average source amplitude of all nodes within an ROI at any given time point. The ROI amplitude data were converted to z-scores timewise to minimize inter-participant variabilities that allowed all ROIs to be equally weighted in the k-means algorithm. Then, the ROI-based z-score time courses from all participants were concatenated as the input to the k-means algorithm (Lloyd, 1982) using correlation as the distance measure for the number of clusters (k) ranging from 10 to 20. The outcome of the algorithm labeled each timeframe to a cluster out of total k clusters based on similarities among individual timeframe data. The participant-level spatial pattern of each CAP (i.e., a cluster) was constructed via averaging of all timeframe data in a participant’s recording labeled as the cluster. The group-level CAPs were calculated by averaging all participant-level spatial maps of the corresponding CAPs. Normalized cortical source data were used to obtain these spatial maps as opposed to the ROI data to maintain high spatial resolutions. Temporal properties of CAPs were defined in two measures, that is, lifetime and occurrence rate. An occurrence of a CAP was defined as multiple consecutive timeframes that were labeled toward the same CAP, and the occurrence rate of a CAP was its total number of occurrences divided by the entire time of recording from each participant. Lifetime of a CAP was calculated for each occurrence as the total time of consecutive timeframes of one occurrence. These two temporal measures were calculated in individual participants and then averaged for group-level statistics. In the subsequent analysis, results of 20 CAPs (k = 20) were used.

### 2.5 Characteristics of CAPs and comparisons with time-averaged RSNs

Obtained CAPs were named according to the resemblance of their spatial patterns to known anatomical regions and time-averaged RSNs reported in the literature (Beckmann et al., 2005; Thomas Yeo et al., 2011). Each CAP was either resolved as a hemisphere-bilateral pattern or as one of a hemisphere-symmetric CAP pair. These symmetrically paired CAPs were named with “left (L)” or “right (R)” after their anatomical names to indicate their dominant side. To facilitate comparisons between large-scale transient patterns, that is, CAPs, and time-averaged RSNs, that is, ICs, the 20 CAPs were matched one-to-one with the 29 neuronal ICs through visual inspections. However, several CAPs showed bilateral patterns while their corresponding ICs were unilateral. In these cases, a single CAP was matched with the pair of hemisphere-symmetric ICs. The same one-to-two matching was applied if an IC was bilateral and its corresponding CAPs were unilateral. To validate the outcome of matching, the similarity measures of spatial correlation and dice score were computed to construct the confusion matrices between all matched/unmatched pairs of CAPs and RSNs. All spatial correlation values were converted to z-scores using the Fisher transform to normalize their distributions. Dice scores were calculated over binarized spatial maps of CAPs and RSNs thresholded at 20% of their corresponding maximal values. For a bilateral CAP matched with two hemisphere-symmetric ICs (or vice versa), both single-hemisphere similarity measures were calculated on left hemisphere and right hemisphere separately. A permutation-based test was performed to evaluate the statistical significances of the two similarity measures from individual matched pairs of CAPs and RSNs against a null distribution of these measures from CAP–RSN pairs randomly created by permuting the labels of RSNs 100,000 times.

Beyond comparing spatial patterns of CAPs directly to RSNs, other spatial and temporal characteristics of CAPs were also evaluated. Firstly, to assess the hemispheric symmetry of CAPs, cortical spatial patterns were averaged into 148 ROIs (74 matching ROIs in each hemisphere) using an alternative yet still well-established cortical surface atlas (Destrieux et al., 2010), which provided a more hemispheric-symmetric parcellation due to its anatomically derived parcellation than the Schaefer’s functionally based parcellation used in prior clustering steps. The hemispheric symmetry index was calculated as the Pearson correlation between the original ROI-based spatial pattern and its mirrored pattern (created by swapping the ROI-based values between the left and right hemispheres). For the unilateral CAP pairs, the original and mirrored ones referred to one of the pairs. For the bilateral CAPs, the mirrored one was from itself. The hemispheric symmetries of RSNs were similarly calculated and used as references to be compared with those of CAPs. Secondly, to assess inter-participant consistencies of CAP spatial patterns, spatial correlations between the participant-level cortical maps and the corresponding group-level cortical maps of each CAP were calculated using a leave-one-out approach, where the group-level maps were computed from all participants other than the participant being compared. The inter-participant consistencies of RSNs were similarly calculated and compared with those of CAPs. For RSNs, it was noted that inter-participant consistencies could be reflected in the magnitude of participant-level beta-values of ICs (see Section 2.3). Therefore, the average beta-value within masked regions defined on the top 5% activated region of group patterns was calculated for each participant’s IC spatial map. The mean beta-value was compared with the inter-participant consistency score. Lastly, the correlations between temporal fluctuations of CAP occurrences and RSN dynamics (i.e., IC time courses) were calculated at each participant and then averaged across all participants. To overcome the mismatch between CAP occurrences (i.e., binary) and RSN dynamics (i.e., continuous), the concept of CAP time courses was adopted (Gutierrez-Barragan et al., 2019), which were obtained by computing the spatial correlation between each CAP and the ROI-based z-scores at each timeframe (see Section 2.4). A permutation-based test was performed to evaluate the statistical significances of temporal measures in the same manner as for spatial measures discussed above.

## 3 Results

### 3.1 Transient CAPs spatially resembling time-averaged RSNs

**Fig. 1.**
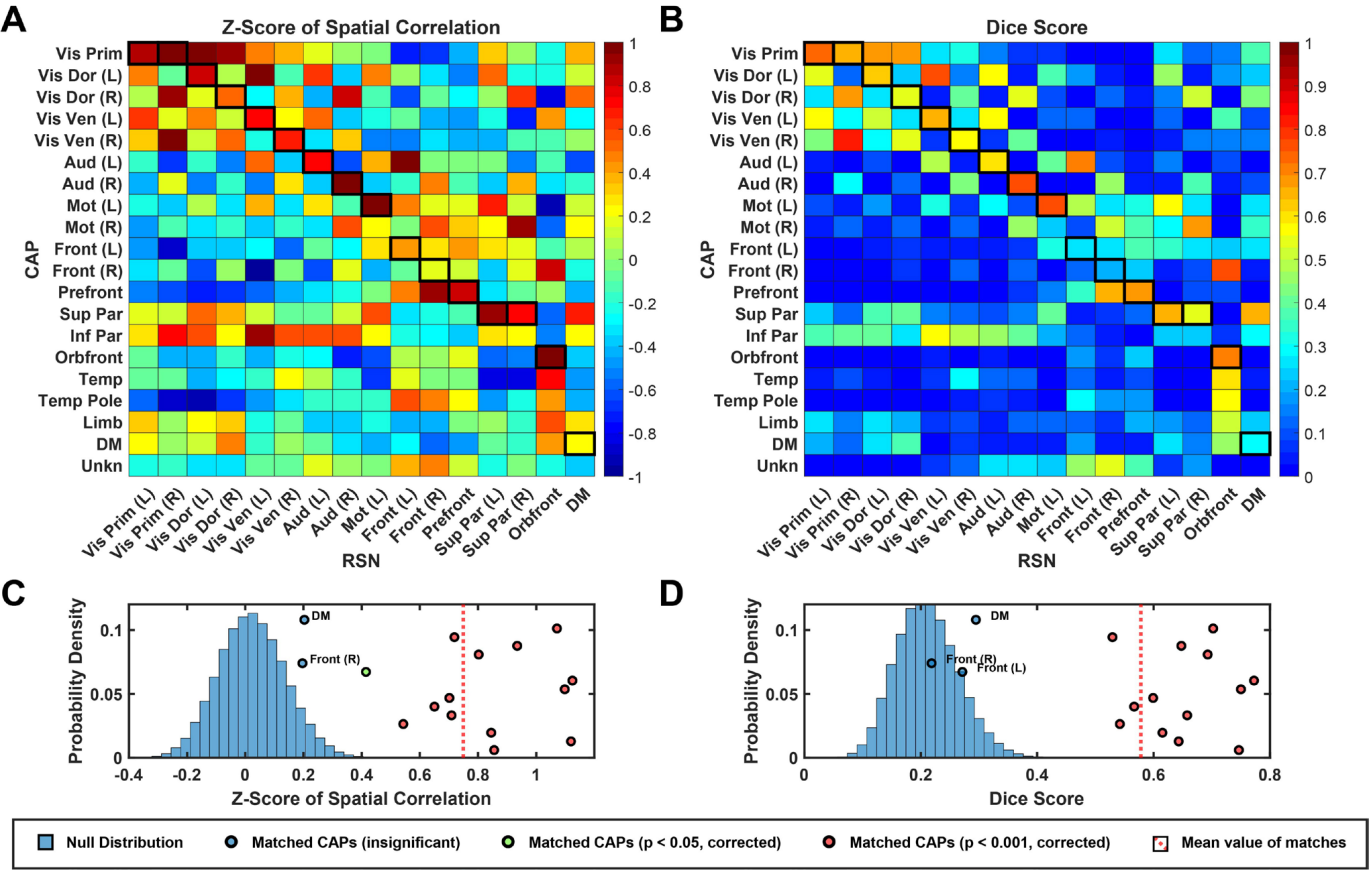
Spatial similarity between CAPs and RSNs. (A, B) The confusion matrix of spatial correlation values (converted to Fisher z-scores) (A) or dice scores (B) between CAPs and RSNs. Matched CAP–RSN pairs are outlined in black. (C, D) Statistical comparisons of two similarity measures, that is, spatial correlation (C) and dice score (D), between the matched CAP–RSN pairs and 100,000 randomly permuted CAP–RSN pairs. Dots: 16 individual matched pairs with statistically significant ones in red (*p *< 0.001, *corrected*) or in green (*p* < 0.05, *corrected*), and insignificant ones in blue. Dashed line: the mean values from all 16 matched pairs of statistical significance (*p* < 0.001).

According to the naming protocol in Section 2.5, 20 CAPs are categorized into 3 groups: Sensorimotor (SM) CAPs, each mainly covering cortical areas responsible for sensory or motor functions; High-Order (HO) CAPs, each mainly covering either frontal, temporal, and/or parietal cortical areas; and Other CAPs, each covering either distributed cortical areas or limbic areas. Out of the total 20 CAPs, 14 CAPs are visually matched with RSNs (Fig. 1A). From the perspective of RSNs, the majority of ICs were also matched with CAPs (18 matched ICs out of total 29 neuronal ICs, Supplementary Fig. S1). It is noted that 11 unmatched ICs in general exhibit lower regression beta-values (indicating lower contributions to data variances and lower inter-participant consistency) than matched ones (Supplementary Fig. S2B) that are of statistical significance (*p* < 0.001, *corrected*, Supplementary Fig. S2D). Among the three ICs matched to the same Vis Dor (R) CAP (Supplementary Fig. S1), only the IC with the highest regression beta-value was selected (motivated by the above observation of low beta-values in the unmatched ICs) for the analysis of confusion matrix, resulting in a total of 16 matched RSNs. Figure 1A and B illustrates the confusion matrix of spatial correlations and dice scores, respectively, between the 20 CAPs and 16 RSNs (total 16 matched pairs with 2 bilateral CAPs each matched to 2 unilateral RSNs). In general, the matched pairs (elements emphasized with black boxes) have higher similarity values than the unmatched pairs (unboxed elements). The average z-score (0.74) and dice score (0.57) of matched pairs were found to be significantly higher (*p *< 0.001) than those from the randomly permuted null distribution of alternative matching combinations, indicating that the group-level CAPs are spatially similar to their matched RSNs. For individual matched pairs, most of them are found to have significantly higher (*p *< 0.001, *corrected*) similarities in both measures than those from the random permutation. Meanwhile, three matched CAPs (i.e., the Front CAPs (L & R) from the HO group and the DMN CAP from Other group) showed weaker or no significance in these two similarities measures. Note that the Front (L) CAP exhibits a weaker yet significant correlation measure at *p *< 0.05 (*corrected*).

**Fig. 2.**
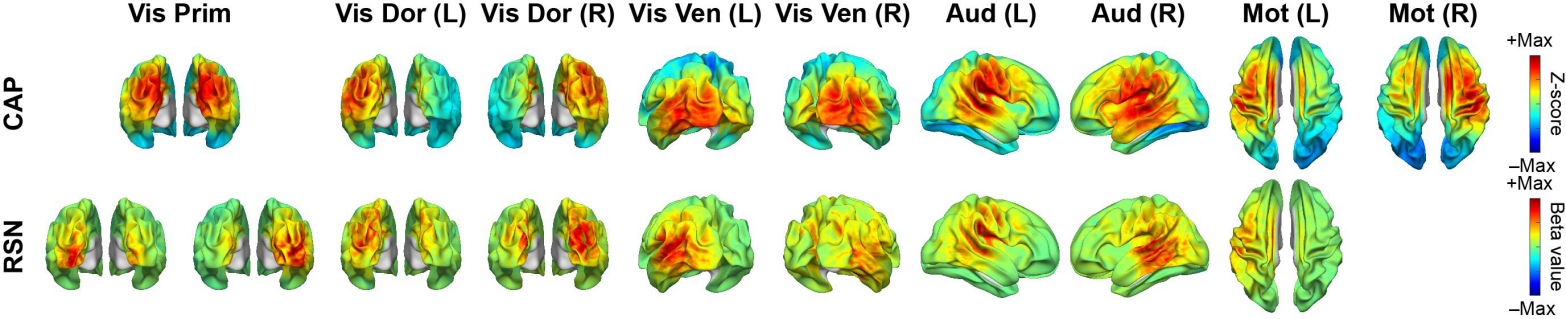
Sensorimotor CAPs and their corresponding RSNs, including primary visual (Vis Prim, matched to two RSNs), visual dorsal stream (Vis Dor), visual ventral stream (Vis Ven), auditory (Aud), motor (Mot, no RSN match for Mot (R)) CAPs. L: left, R: right.

**Fig. 3.**
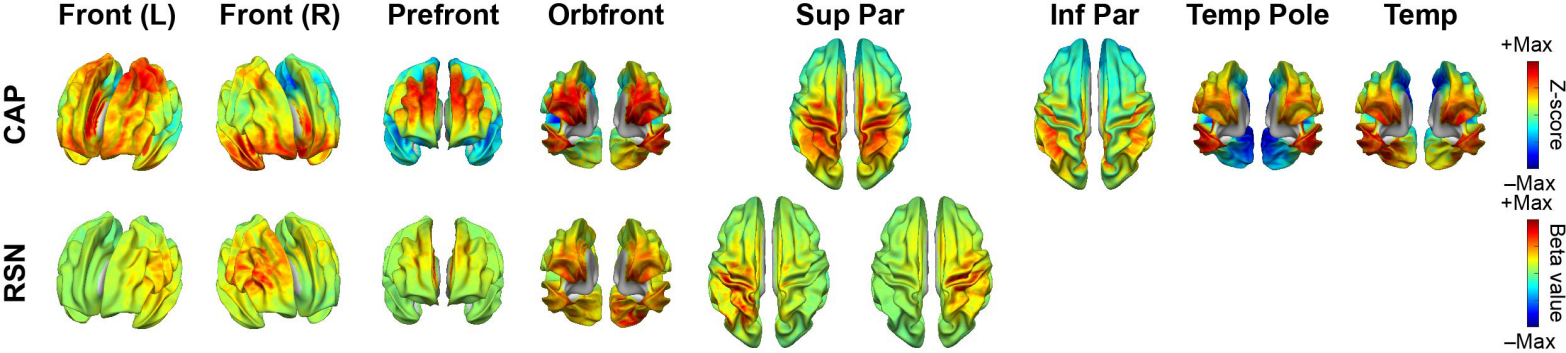
High-order CAPs and their corresponding RSNs, including frontal (Front), prefrontal (Prefront), orbital frontal (Orbfront), superior parietal (Sup Par, matched to two RSNs), inferior parietal (Inf Par, no RSN match), temporal pole (Temp Pole, no RSN match), and temporal (Temp, no RSN match) CAPs.

**Fig. 4.**
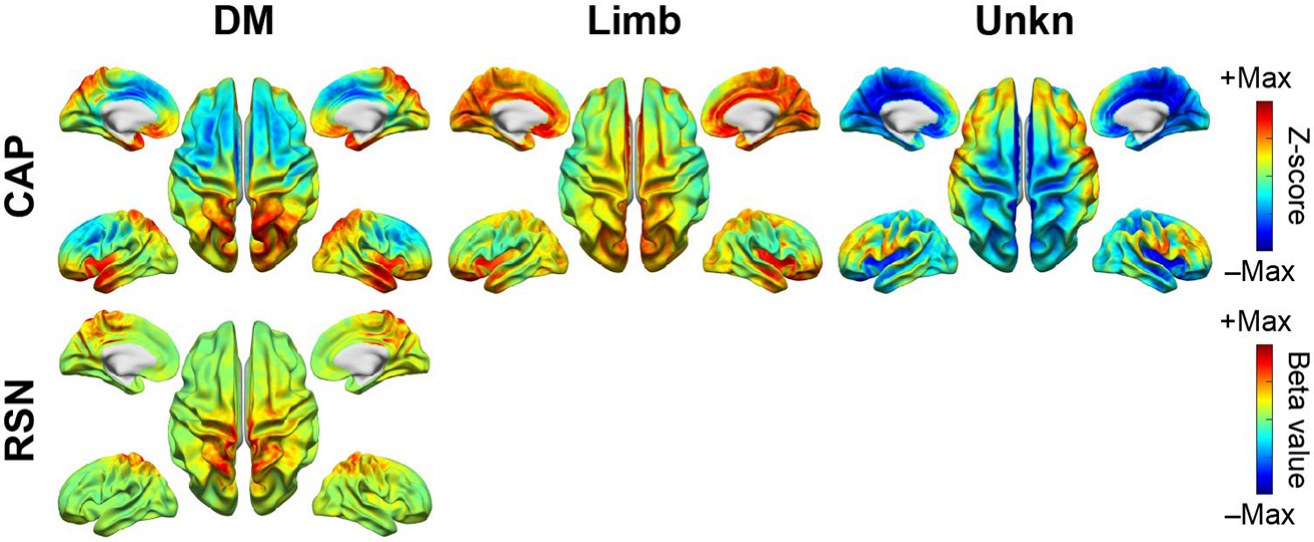
Other CAPs and their corresponding RSNs, including default mode network (DM), limbic (Limb, no RSN match), and unknown (Unkn, no RSN match) CAPs.

The spatial similarities between matched CAPs and RSNs can also be visually observed directly in their spatial maps. Figure 2 illustrates SM CAPs and their matched RSNs, which anatomically encompass the primary visual network (Vis Prim), dorsal visual stream (Vis Dor), ventral visual stream (Vis Ven), auditory (Aud), and sensorimotor (Mot) networks. Figure 3 illustrates HO CAPs and their matched RSNs, which anatomically cover the frontal (Front), prefrontal cortex (Prefront), orbital frontal (Orbfront), superior parietal (Sup Par), inferior parietal (Inf Par), temporal pole (Temp Pole), and temporal (Temp) regions. Figure 4 illustrates Other CAPs, which exhibit spatial patterns similar to DMN and limbic network (Limb), as well as an unknown network (Unkn) that hemispheric-symmetrically runs along the inferior frontal sulcus, cuts through the sensorimotor cortex above the lateral fissure, and then extends the occipito-parieto-temporal junction. Among these CAPs, the corresponding RSN is only identified in the DMN CAP. It is observed via comparing Figures 2, 3, and 4 that SM CAPs display notable similarities to their RSN counterparts while the spatial correspondence of HO and Other CAPs to their matched RSNs is less distinct. This observation is further confirmed by the quantitative correlation values in the confusion matrix (Fig. 1A), where the boxed elements on the left top quadrant (for SM CAPs) show higher values generally than the boxed elements on the right bottom quadrant (for both HO and Other CAPs). Furthermore, the six CAPs that are not matched include one from the SM CAP group, that is, Mot (R) CAP, and five from both the HO and Other CAP groups, that is, Inf Par, Temp, Temp pole, Limb, and Unkn CAPs. The hemispheric counterpart for the Mot (R) CAP, that is, the Mot (L) CAP, is matched with an RSN with a high spatial correlation value (i.e., 0.79).

### 3.2 Hemispheric symmetries of CAPs

**Fig. 5.**
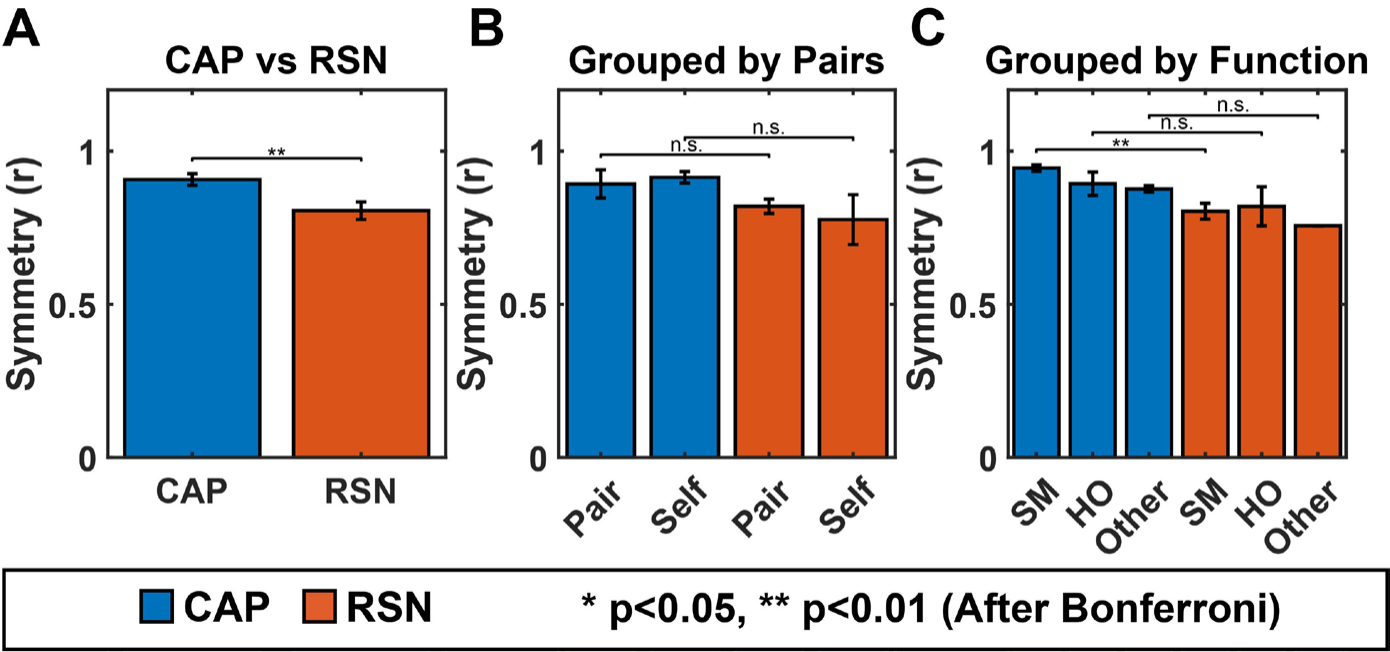
Hemispheric Symmetries of CAPs and RSNs. The bar plots display the hemispheric symmetry (r) values of CAPs and RSNs grouped by (A) overall; (B) symmetric pairs (one L CAP/RSN and one R CAP/RSN) or single bilateral CAPs/RSNs (self); and (C) by three groups (SM, HO, and Other groups). Error bars: standard errors. n.s.: non-significant.

The cortical maps of CAPs exhibit distinct spatial patterns of hemispheric symmetries, which are presented either as pairs of left- and right-dominant unilateral CAPs or individual bilaterally symmetric CAPs. Among the 20 identified CAPs, 10 CAPs are presented as unilateral CAP pairs (i.e., Vis Dor, Vis Ven, Aud, Mot, and Front), while the remaining 10 CAPs are presented as individual bilateral CAPs (Vis Prim, Prefront, Sup Par, Inf Par, Orbfront, Temp, Temp Pole, Limb, DM, Unkn). The quantitative measure of symmetric index supports the strong hemispheric symmetries of CAP cortical maps with the score of 0.89±0.04 for unilateral CAP pairs and the score of 0.91±0.01 for individual bilateral CAPs (Fig. 5B). It is also observed that both HO and Other CAPs appear more often as bilateral spatial patterns than SM CAPs. Only one of nine SM CAPs (i.e., Vis Prim) is bilateral, compared with the six out of eight HO CAPs and the three of three Other CAPs that show bilateral patterns. The cortical maps of RSNs also exhibit hemispheric symmetries while a smaller number of RSNs have bilateral symmetric patterns on both hemispheres (3 out of the 16 ICs). Most of RSNs (13 of the 16 ICs) appear as pairs of symmetric unilateral CAPs (with the exception of Mot L that was not paired). In comparison with CAPs, the symmetric index values for RSNs significantly decrease overall (*p* < 0.01, *corrected*) (Fig. 5A) and in the subset of SM CAPs and RSNs (*p* < 0.01, *corrected*) only (Fig. 5C).

### 3.3 Inter-participant consistencies and varibilities of CAP spatial patterns

**Fig. 6.**
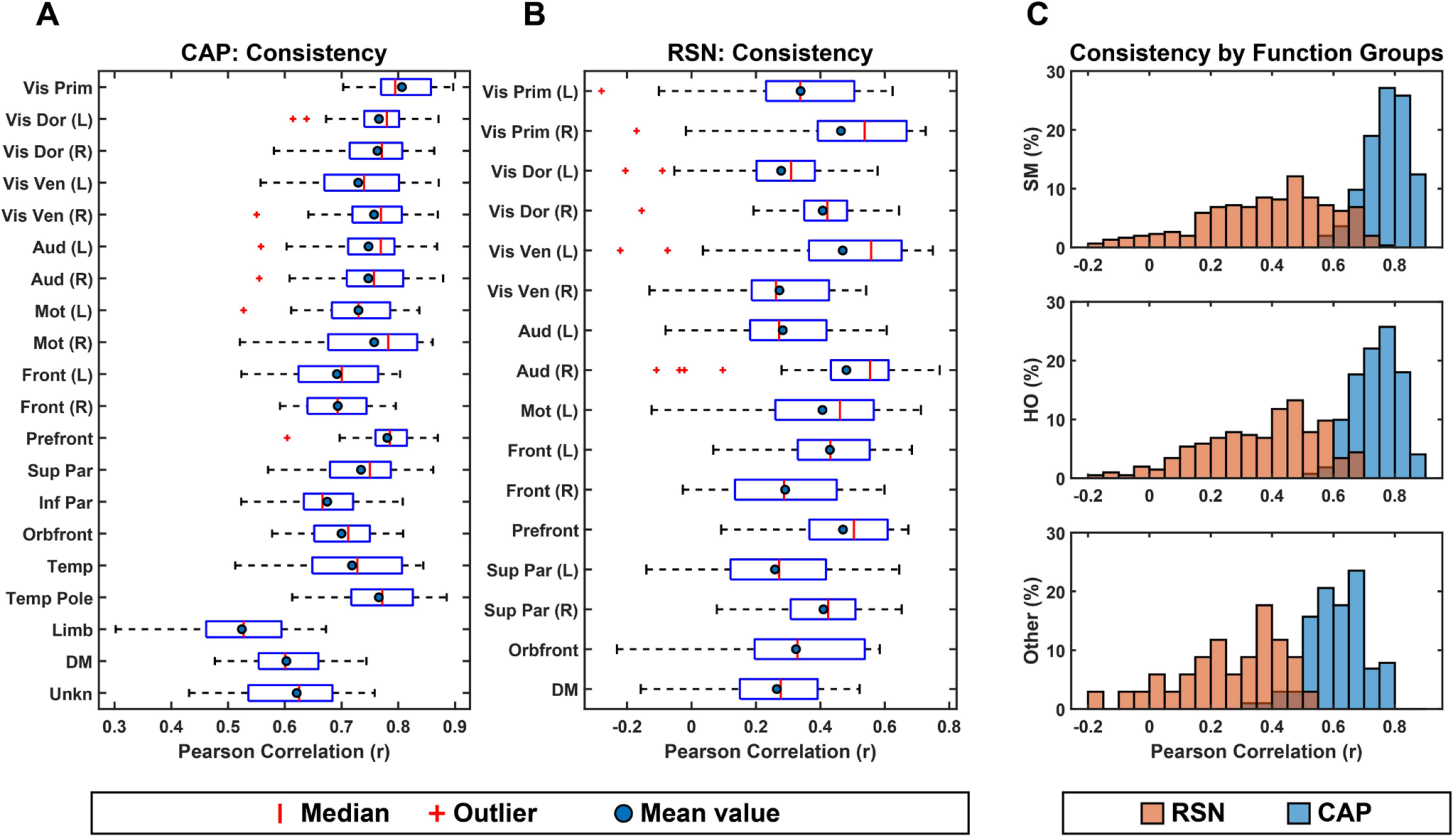
Inter-participant consistency in spatial patterns of CAPs and RSNs. (A) The boxplots of inter-participant consistency measure for 20 CAPs. (B) The boxplots of inter-participant consistency measure for 16 RSNs. Red bars: median; red “+”: outliers greater than the 1.5 interquartile range of the box, blue dots: mean. (C) Histograms of inter-participant consistency measures for the three groups of CAPs and RSNs (SM, HO, and Other). The y-axis has been converted to percentages to account for the disproportionately lower number of Other CAPs than the other two groups.

Figure 6A shows the boxplots of the inter-participant consistency measures based on spatial correlation. In general, CAPs display high inter-participant consistencies with their median spatial correlations close to or higher than 0.65 (the majority close to or higher than 0.75) with only one exception (close to 0.55 for the Limb CAP). In contrast, the inter-participant consistencies of RSNs are lower (Fig. 6B), as all their median spatial correlations are close to or below 0.5. However, it is important to note that the different inter-participant consistencies between CAPs and RSNs might be partially attributed to the methodological differences in reconstructing them in individual participants (see Sections 2.3 and 2.4). More interestingly, such spatial consistency measures exhibit certain variabilities among three CAP groups, in which, relatively speaking, the SM CAPs have the highest inter-participant consistencies, the HO CAPs are in the middle, and the Other CAPs have the lowest consistencies. This relative pattern can be easily observed in the histograms of spatial correlation values for the CAPs from these three groups (Fig. 6C). These histograms illustrate that the spatial correlations for SM CAPs have both the highest values and the narrowest distribution, indicating less variabilities while the spatial correlations for Other CAPs have the lowest values and relatively widest distribution, indicating more variabilities. The relatively moderate values and distribution of the spatial correlations from HO CAPs indicate their higher variabilities than SM CAPs, but lower variabilities than Other CAPs. Similar observations could be partially observed among three corresponding RSN groups in their histograms (Fig. 6C).

To examine the group differences quantitatively, a mixed-effect linear model was used on all z-scored correlation values from both CAPs and RSNs with the fixed effect of pattern (CAPs vs RSNs) and group (SM, HO, and Other groups) and a random effect for individuals. Results of the mixed-effect model revealed significantly higher (*p* < 0.001) inter-participant consistency of CAPs than RSNs across all three groups, significantly higher (*p *< 0.05) consistency of SM groups than HO groups in CAPs but not in RSNs, significantly higher (*p *< 0.001) consistency of HO groups than Other groups in CAPs and RSNs, and significantly higher (*p *< 0.001) consistency of SM groups than Other groups in both CAPs and RSNs. With one exception (SM vs HO in RSNs), a general pattern of the highest consistency among the SM group and lowest consistency among the Other group is conserved in both CAPs and RSNs. Furthermore, RSNs with higher mean beta-values (Supplementary Fig. S2B) from all participants generally showed great inter-participant consistency of spatial patterns (Supplementary Fig. S2A), which is supported by the positive correlation (r =0.59 , p<0.05) between the beta-value magnitudes and inter-participant consistencies from all participants (Supplementary Fig. S2C).

### 3.4 Recurring transient patterns of CAPs

**Fig. 7.**
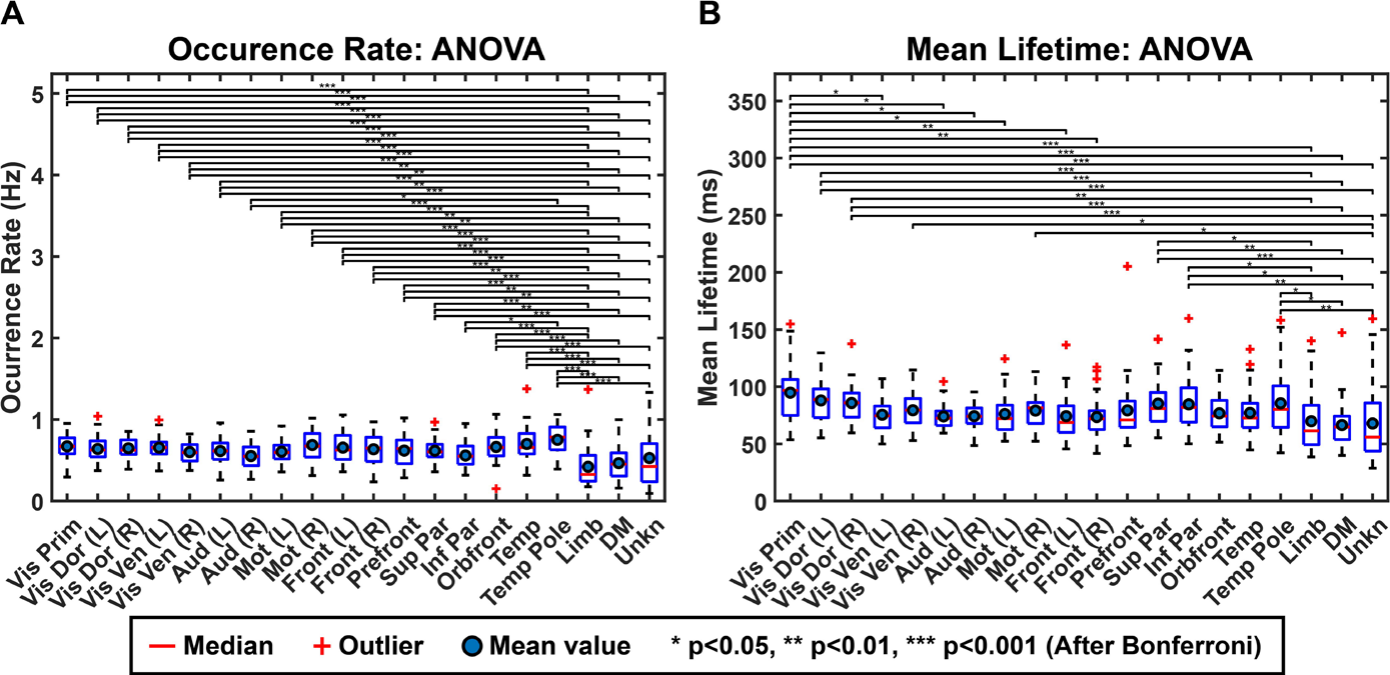
Boxplots of the occurrence rate (A) and mean lifetime (B) for 20 CAPs. Differences were assessed using ANOVA and then post hoc t-test on the log-transformed values of occurrence rate and lifetime and statistical significances were corrected using the Bonferroni correction for total 190 pairs.

While CAPs show high similarities to time-averaged RSNs in spatial patterns, CAPs temporally are more characterized as recurring transient events. The lifetimes of all 20 CAPs, averaged across all participants, are consistently in the range 66–94 ms (Fig. 7B), demonstrating the sub-second, transient nature. These lifetime data suggest that resting human brains rapidly transition among a set of configurations dominated by various functional systems (i.e., SM, HO, and Other CAPs) rather than dwelling in any one configuration for long time. At the same time, all CAPs recur at the average rates lower than 1 Hz, each of which remain largely consistent across participants (largely varies between 0.5 and 0.9 Hz except the three Other CAPs). It is also noted that all 20 CAPs were present in all individual participants. These observations indicate that these CAP patterns emerge intermittently, and the brain states represented by them are being visited in a repeatable manner over time.

Among the three groups, the Other CAPs exhibit the largest variabilities in both mean lifetime and occurrence rate among individuals (Fig. 7). One-way ANOVA on the log-transformed occurrence rates and mean lifetimes performed over all possible pairs of 20 CAPs revealed that 47 out of total 49 statistically significant differences (*p* < 0.05, *corrected*) in occurrence rate and 20 out of total 26 statistically significant differences (*p* < 0.05, *corrected*) in mean lifetime observed are associated with the 3 Other CAPs. Moreover, significant differences for occurrence rate at *p* < 0.01 (total 47) and mean lifetime measures at *p* < 0.001 (total 9) observed are all associated with the 3 Other CAPs. These significant differences indicate lower occurrence rates of these three CAPs than most of other CAPs, with the Limb CAP exhibiting the lowest occurrence rate (Fig. 7A). The results for lifetime indicate shorter mean lifetimes of these three CAPs than all other CAPs, but the difference only reaches statistically significant levels with three visual CAPs, that is, the Vis Prim and Vis Dor (L and R) CAPs, which have the longest lifetimes (Fig. 7B). These three CAPs further exhibit increased inter-participant variability compared with all other CAPs (e.g., increased heights of boxes in Figure 7A and B indicating the difference between 75^th^ percentile and 25^th^ percentile).

**Fig. 8.**
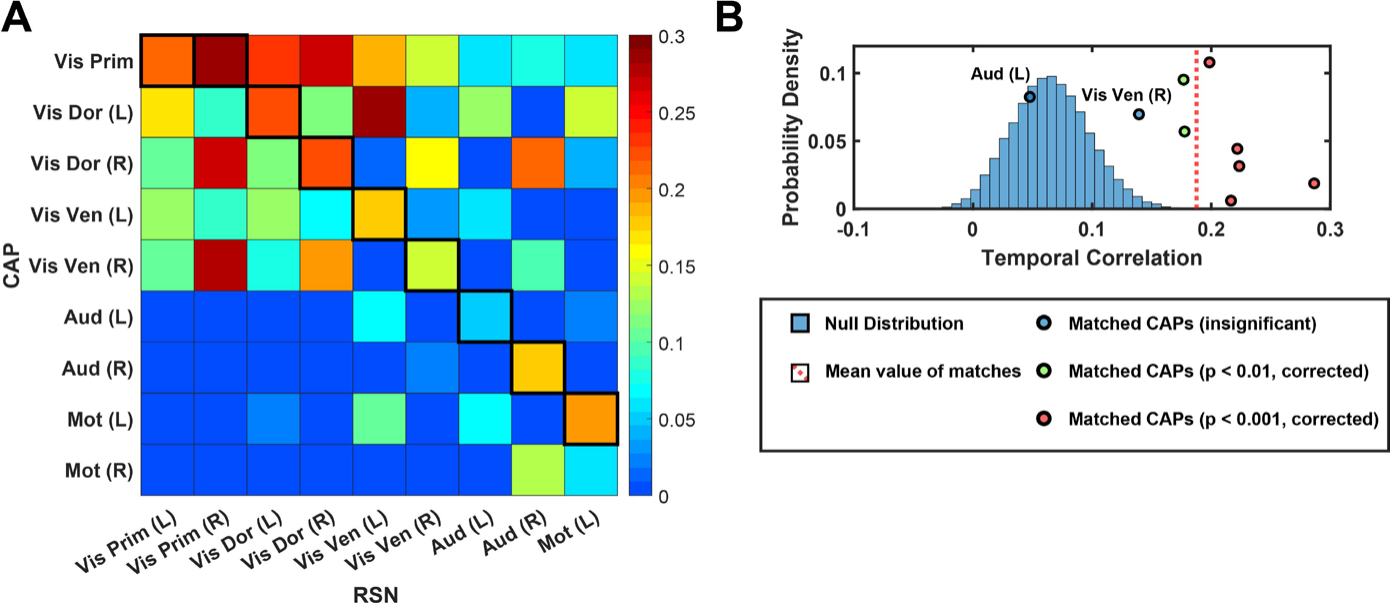
Temporal fluctuation correspondences between CAPs and RSNs from the SM group. (A) The confusion matrix of temporal correlations (converted to Fisher z-scores) between CAPs and RSN. (B) Statistical comparisons of temporal correlation between the matched CAP-RSN pairs and 100,000 randomly permuted CAP–RSN pairs. Others figure elements are illustrated the same as in Figure 1.

Temporal fluctuation correspondences between CAP occurrences and RSN dynamics were only investigated for those from the SM group that were best matched spatially (Fig. 1) and consistently across participants (Fig. 6) due to the mismatch in their temporal measures (i.e., binary vs continuous) and the introduction of a surrogate temporal activation measure for CAPs (i.e., CAP time series, see Section 2.5). The average z-scored temporal correlation (0.18) of matched pairs was found to be significantly higher (*p *< 0.001) than those from the randomly permuted null distribution (Fig. 8B), indicating that, in general, most CAPs exhibit similar temporal fluctuation to their matched RSNs at individual participants (Fig. 8A). This observation is also supported by the fact that most individual matched pairs (seven out of nine) were found to have significantly higher (at least *p *< 0.01, *corrected*) temporal fluctuation similarities (Fig. 8B).

### 3.5 Reproducibility of CAPs with different clustering parameters

**Fig. 9.**
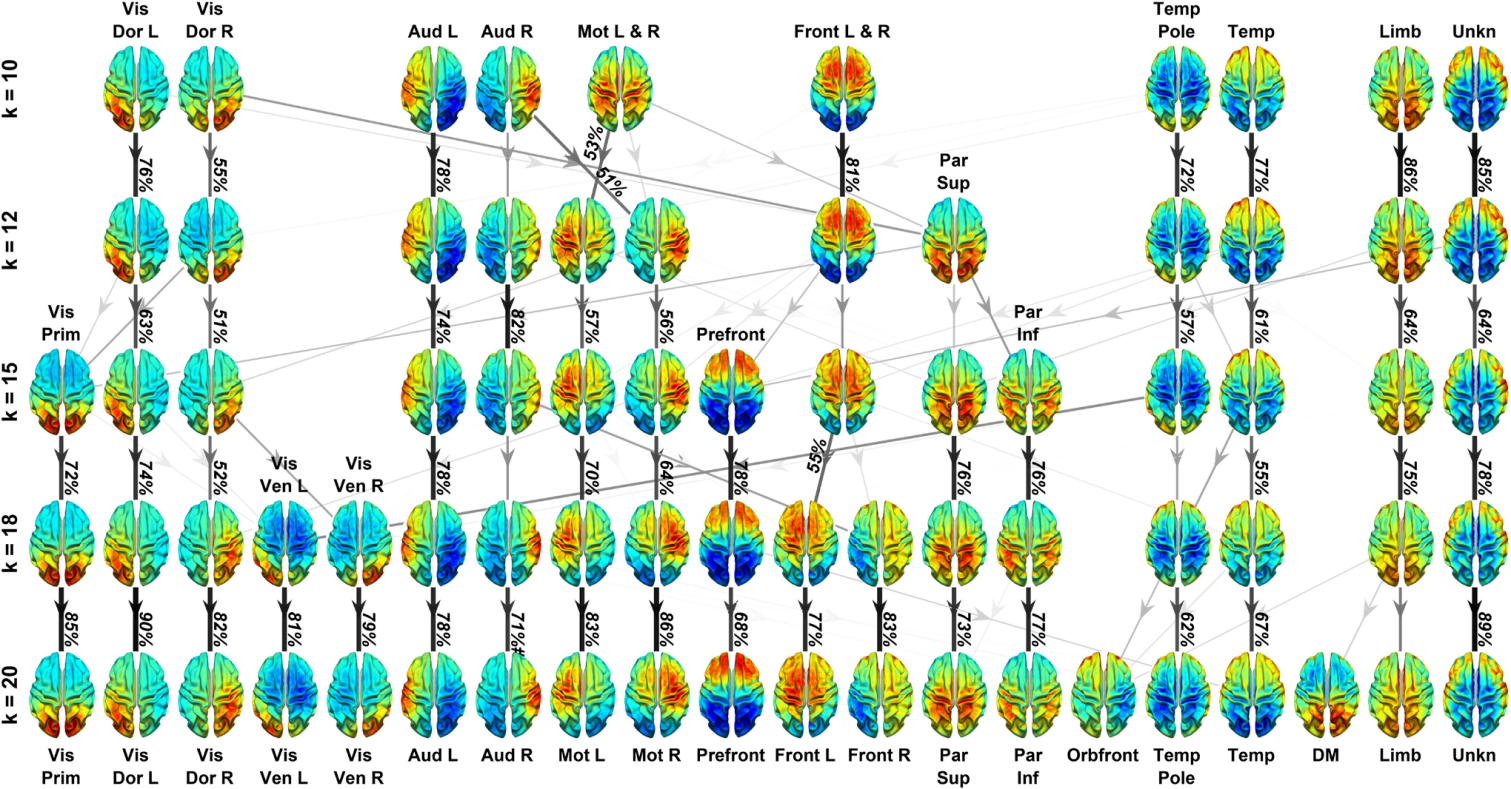
Graph showing the correspondences of CAPs at different k values and their timeframe overlaps. Each arrowed edge indicates the percentage of timeframes belonging to a CAP at a k value that are overlapped with a CAP at the next-higher k value. Edge thickness and darkness are coded with the percentage values. For visual clarity, only edges with the percentage values >10% are displayed, and labeled for those >50%.

Beyond the reproducibility of CAP’s spatial (Fig. 6A) and temporal patterns (Fig. 7) among individuals, the reproducibility of CAPs due to pre-selected parameters for the k-means algorithm, that is, the number of clusters (k), was investigated. Figure 9 presents a graph that illustrates the group-level cortical maps of CAPs as the k value increases from 10 to 20 with the number on each arrow indicating the percentage of timeframes of a CAP at a k value overlapped to a CAP at the next-higher k value. In general, identified CAPs at different k values remain spatially consistent, demonstrating their reliable detections even when the k value changes in a relatively wide range (i.e., doubled). When k increases, additional clusters that are spatially close emerge from the existing CAPs at lower k values. Some of these newly emerged CAPs reflect finer sub-systems within the same functional system. An example of the hierarchical subdivision via splitting is the Mot CAP, which shows a bilateral pattern over two hemispheres at k = 10 and splits into a left-hemisphere-dominant and a right-hemisphere-dominant CAP at k = 12. The Vis Prim CAP starts to appear when k = 15, which merges the splits from both left and right Vis Dor CAPs. Both the left and right Vis Ven CAPs emerge at k = 18 from splits of the left and right Vis Dor CAPs and the Vis Prim CAP at k = 15. It is also noted that some new CAPs further emerge at the highest k value (i.e., k = 20), which might suggest that further investigations into even higher k values (>20) might be still valuable. For example, the Orbfront CAP only appears at k = 20, which emerges with the splits mainly from the Temp, Temp Pole, and Limb CAPs due to its spatial similarities in the frontal cortex. It is also interesting to note that among three Other CAPs, both the Limb CAP and Unkn CAP are consistently present from k = 10 to 20 while the DMN CAP only shows up when k = 20, a split mainly from the Limb CAP. These examples not only indicate the persistence of these patterns across different k values but also suggest that CAPs are not merely artifacts of a specific clustering choice but instead reflect functional systems that can be resolved at multiple levels of granularity.

In addition to the value of k, CAP reproducibility was observed with different clustering seed initializations in four additional iterations of the CAP analysis at k = 20. In all iterations, the same labeled set of 20 CAPs emerged, with spatial patterns remaining stable in all but some minor variations in the Front (L, R) and Limb CAPs (Supplementary Fig. S3). The Limb CAP alternates between localizing to the cingulate cortex and extending to broader areas on the middle wall across iterations. The Front CAPs vary slightly in their degree of symmetry between the left and right hemispheres across iterations. Sensorimotor and DMN CAPs, by contrast, show no noticeable variations across iterations (data not shown).

## 4 Discussion

In the present study, we identified a set of reproducible recurring and transient co-activation patterns (CAPs) in EEG that spatially resemble canonical time-averaged RSNs. Beyond spatial matches, CAPs exhibit RSN-like characteristics, including hemispheric symmetry, separatable granularity in functional systems, intersubject variability gradient across low-level and high-level functional systems, and the existence of both DMN and TPNs. These findings suggest that time-averaged RSNs, constrained due to the use of time-domain measures (e.g., temporal correlation and independence), might be expressions of brief, recurring, spatially large-scale neuronal events. On the contrary, CAP analysis utilizing spatial-domain measures can resolve such spatially large-scale neuronal events at much finer temporal scales (<100 ms), which potentially offers a new window on investigating fast dynamics of such large-scale neuronal events.

### 4.1 Spatial patterns of CAPs

Our findings provide the evidence that cortical CAPs reconstructed from EEG data exhibit strong spatial similarities to time-averaged RSNs that have been widely reported in fMRI literatures (Beckmann et al., 2005; Damoiseaux et al., 2006). Nine SM CAPs recover distributed cortical co-activation patterns within the somatosensory, visual, auditory, and motor systems. For the visual system, the existence of multiple CAPs indicates that the primary, ventral, and dorsal visual cortices are spatially separatable in the functional networks defined by CAPs. Two unilateral motor CAPs reveal symmetric but opposite hemisphere-dominant distributed activations each involving both the primary motor cortex and supplementary motor areas (Fig. 2). The DMN CAP reveals the full membership of cortical nodes of DMN established in fMRI within the medial prefrontal cortex, posterior cingulate cortex, temporo-parietal junction, and temporal lobes on both hemispheres. Several HO CAPs (left and right Front, Prefront, and Orbfront CAPs) cover the areas for executive control networks identified in fMRI (Beckmann et al., 2005). Beyond the fMRI literature, the spatial patterns of these CAPs are also similar to time-averaged RSNs found in EEG data (Liu et al., 2017; Yuan et al., 2016) and MEG data (Brookes et al., 2011; De Pasquale et al., 2010; Hipp et al., 2012), which is not surprising as the spatial correspondences between EEG RSNs and fMRI RSNs have been reported (Yuan et al., 2016). Such similarities are also investigated in the present study, and our results indicate the strong correspondences between CAPs and RSNs derived from the same EEG dataset (Figs. 2–4), which is supported by high values of two spatial similarity measures (both of statistical significance) averaged over all matched CAP–RSN pairs as well as in individual pairs (most of statistical significance) (Fig. 1C, D). The similarities between CAPs and RSNs are not only observed among individual matched pairs, but also collectively among the entire identified sets of CAPs. In the present study, of the 20 CAPs identified, 14 CAPs show high spatial similarities to RSNs reconstructed from the same dataset. Compared with the fMRI literature, the only frequently reported network patterns missing from the set of CAPs are the frontoparietal RSNs (Damoiseaux et al., 2006), which are however also often missed in EEG/MEG RSNs (Liu et al., 2017). Considering the detections of both frontal and parietal CAPs (Fig. 3), a potential hypothesis is that these networks might prevail in different frequency ranges other than the alpha band investigated in the present study as large-scale synchronizations are frequency specific (Hipp et al., 2012; Vidaurre et al., 2018). The present study also produces a CAP (i.e., the Unkn CAP), which does not resemble any known RSNs. It might be the moment-to-moment snapshots of brain states with the most representative spatial patterns along the dorsal-ventral propagating waves (Majeed et al., 2011) as similarly reported in hemodynamic signals (Khan et al., 2022).

The present study also provides evidence that suggests the correspondence between CAPs and RSNs beyond their matching spatial patterns. Firstly, EEG CAP spatial patterns display high hemispheric symmetry (Fig. 5) and strong hemispheric symmetries are the hallmark of RSNs (Smith et al., 2009) and other network patterns from fMRI such as resting-state functional connectivity (Calhoun et al., 2014), CAPs (Gutierrez-Barragan et al., 2019; Liu et al., 2013), and large-scale propagation waves (Matsui et al., 2016; Mitra & Raichle, 2016). Such hemispheric symmetries are also observed in EEG/MEG (Takeda et al., 2021; Yuan et al., 2016) and fNIRS studies (Khan et al., 2021, 2022, 2024) regarding RSNs, CAPs, and other large-scale resting-state activations and/or phenomena, as well as in the EEG RSNs from the present study (Fig. 5). Secondly, the functional systems represented by CAPs could be separated into multiple levels of granularity depending on chosen clustering parameters. For examples, varying the k value from 10 to 20 illustrates the process of generating CAP representations for more sub-systems within both the visual cortex and the motor system (Fig. 8). These observations are consistent with hierarchical anatomic organizations of human cortical structures (Thomas Yeo et al., 2011). Their separatable representations in functional data have been reported in fMRI where more RSNs appear to represent sub-systems when the complexity of model increases, for example, the number of ICs (Smith et al., 2009). Lastly, increased inter-participant variability is observed from SM CAPs to HO CAPs (Fig. 6A, C), which is consistent with intersubject variability gradients (Mueller et al., 2013; Seitzman et al., 2019) across lower-order perceptual networks (lower inter-participant variability) and higher-order functional networks (higher inter-participant variability). These observations suggest that the spatial patterns of CAPs are modulated in the same direction as RSNs on three important properties of functional neural systems, that is, homologue, sub-systems, and individual variability, therefore, indicating possible common distributed neuronal substrates behind both CAPs and RSNs.

Several large-scale patterns of transient nature (at similar timescales as CAPs) have been identified from EEG/MEG, that is, microstates (Britz et al., 2010; Custo et al., 2017; Pascual-Marqui et al., 2014), HMM (Baker et al., 2014), and CAPs based on different distance measures, for example, L1-norm (Shou et al., 2022), and different parcels, for example, IC-based functional parcels (Ding et al., 2022). Many of them have been interpreted in the context of RSNs based on their spatial similarities to RSNs. For example, convolving time courses of microstates with hemodynamic response functions (HRFs) have yielded RSN-like spatial patterns (Britz et al., 2010). Similarly, HMM-derived brain states from MEG also resemble canonical RSNs (Baker et al., 2014). However, differences are also observed among these transient patterns. Traditional microstate analyses often only resolve four dominant patterns (Michel & Koenig, 2018) each with a dipolar topography that is usually generated by focal regional activations. Moreover, cortical activation patterns identified with microstate and HMM analyses have been indicated with significantly different spatial patterns (Coquelet et al., 2022). L1-norm distance-based CAPs reveal unique global coactivation patterns that have not been reported in any other transient large-scale phenomena (Ding et al., 2022). When data from a few IC-based functional parcels (i.e., 14 ICs) are used for clustering, L1-norm distance-based CAPs also reveal several RSN-like configurations (e.g., visual, sensorimotor, and DMN networks) beyond global coactivation patterns (Ding et al., 2022). However, when data from a relatively large number of anatomical cortical parcels (i.e., 100 ROI) are used, L1-norm distance-based CAPs do not resemble RSN patterns, instead reconstructing an entire set of global coactivation patterns at different magnitude levels (Shou et al., 2022). Such similarities and differences among various sets of transient brain-wide activation patterns discovered in L1-norm and correlation distance-based EEG-derived CAPs might be explained due to the existence of multiple ongoing large-scale neuronal activations and the varying sensitivities of different models/parameters to different activations (see more methodological discussions in Section 4.5).

### 4.2 Temporal characteristics of CAPs

EEG CAPs obtained in the present study exhibit two distinct temporal characteristics: transience and recurrence. Brain states represented in CAPs manifest themselves as recurring transient episodes of large-scale co-activations between 60 and 100 ms across all participants. All brain states (i.e., 20 CAPs) are visited by every participant, with stable occurrences and lifetimes across all participants and CAPs.

Prior work in EEG/MEG supports the presence of transient brain states lasting on the order of tens to hundreds of milliseconds. Microstate analyses have repeatedly shown that EEG topographies remain quasi-stable for durations below 200 ms (Pascual-Marqui et al., 2014; Custo et al., 2017). HMM analyses on MEG data identify short-lived states on the order of 50–200 ms (Baker et al., 2014; Vidaurre et al., 2018). L1-norm based CAPs exhibit their lifetimes between 25 and 40 ms (Ding et al., 2022; Shou et al., 2022). While variations exist in various large-scale transient neuronal activations, their timescales are consistent with neuronal events revealed in evoked brain activations (tens to hundreds of milliseconds) (Light et al., 2010). Therefore, these lifetime values are potentially indicative of the actual lifetimes of underlying neuronal events, adding new understanding to large-scale networked activations beyond time-averaged RSNs. It is also noted that the selection of approach parameters might impact this temporal metric as well. Our results indicate that, as the k value increases from 10 to 20, the mean lifetimes of CAPs decrease from 102–129 to 66–94 ms. Furthermore, all large-scale transient neuronal activations discussed above (i.e., microstates, HMM, and CAPs with various distance measures) exhibit recurring behaviors.

Our results further suggest that the CAPs from the Other group show significantly shorter lifetimes and lower occurrence rates than many CAPs from both the SM and HO groups. Such significant differences are not observed among any pair of CAPs from within either the SM or HO group or from across these two groups. Shortened lifetimes and lowered occurrences of the Other CAPs might lead to their insufficient and unequal representations within the total 10-minute recordings in individual participants that can explain their significantly higher inter-participant variabilities of spatial patterns (Fig. 6A). Furthermore, the inter-participant variabilities of these two temporal measures in the Other CAPs seem also relatively higher than many CAPs from both the SM and HO groups (Fig. 7), which supports the higher inter-participant variabilities of these CAPs. As the three Other CAPs indicate either highly networked activations or activations outside sensory and motor cortices, these observations are consistent with intersubject variability gradients (Mueller et al., 2013; Seitzman et al., 2019) across lower-order perceptual networks and higher-order functional networks as discussed above.

### 4.3 Relationship between CAPs and RSNs

Our results and observations seem to support the hypothesis that the canonical time-averaged RSNs may stem from temporal aggregations of repeated transient episodes of co‑activations (i.e., CAPs). Firstly, the majority of CAPs and RSNs display significantly similar spatial maps (Figs. 1–4). The CAPs that could not be spatially matched to RSNs typically exhibit shorter lifetimes, lower occurrences, and greater inter-participant variability (e.g., the Other CAPs in Figs. 6 and 7), all of which reduce the formation of spatially and temporally stable patterns over entire recording windows in individual participants and across all participants, thereby potentially limiting corresponding reliable component extraction by the temporal ICA. Secondly, while individual CAPs seem transient, they consistently recur throughout the entire recording window and all participants. Their repeated activations provide temporally sustained components that might lead to their presences as time-averaged RSNs. This is further supported by the observations that the CAPs with high occurrences (e.g., visual, auditory, temporal) are better spatially matched to time-averaged RSNs, whereas the CAPs with low occurrences (e.g., the Limb, Unkn CAPs) are less spatially matched or not matched (Fig. 1A, B). Lastly, both CAPs and RSNs largely show hemisphere-symmetric patterns (Figs. 2–5) and similar inter-participant variability gradients (Fig. 6C) that offer additional evidence about their potential same underlying neural generators. The hypothesis between CAPs and RSNs is supported by several reported fMRI findings. Liu and Duyn (2013) show that averaging the top 1–15% of timeframes based on fMRI BOLD signal amplitudes in pre-selected seeds produces spatial maps highly similar to RSNs derived from a static temporal correlation analysis. Related work further emphasizes that RSN structures could be recovered from high-activation time points alone, without requiring stationary assumptions (Gutierrez-Barragan et al., 2019; Tagliazucchi et al., 2012). While fMRI and EEG are in extremely different timescales, these literatures support the hypothesis that long-timescale neuroimaging patterns may emerge from the recurrence of short-lived, structured brain events. If the hypothesis that CAPs offer a time‑resolved perspective on the large‑scale functional architecture behind RSNs is true, the detection of CAPs would provide insights on networked brain activations at the actual timescale of neuronal events (tens to hundreds of milliseconds) with spatial patterns at a resolution similar to canonical time-averaged RSNs. Such constructs are promised to reveal unsurpassed spatiotemporal dynamics of functional brain activations that can lead to better understanding of cognition (Sadaghiani & Kleinschmidt, 2013) and better predictors of brain disorders (Jin et al., 2017).

### 4.4 Reproducibility

CAPs are consistently reproduced as stable patterns across a range of clusters (i.e., k = 10–20). It also meets the expectation that increasing the number of clusters results in revealing more hierarchical structures in neural systems (e.g., the visual system in Fig. 9), which is the phenomenon similarly observed in fMRI data as discussed above. CAPs are also consistently reproduced using randomly generated seeds initiating the iterative k-means clustering algorithm (Supplementary Fig. S3). Again, sensorimotor CAPs show much less noticeable variations with different initiating seeds than HO CAPs, consistent with their low inter-participant variability and robust group-level presence. The reproducibility is also attested in relatively low-to-moderate inter-participant variabilities in the spatial patterns (Fig. 6A) and temporal measures (Fig. 7) of all CAPs. It is noted that, in comparison with SM and HO CAPs, the Other CAPs are less consistently detected across participants. However, despite occurring less frequently, lasting much shorter, appearing less consistently across participants, and in general accounting for a much smaller variance in EEG data, two of the Other CAPs (e.g., Limb and Unkn) exhibited highly reproducible spatial structures across clustering iterations, appearing as early as k = 10 (Fig. 9).

### 4.5 Methodological considerations and limitations

The key methodological difference between the approaches in reconstructing RSNs and CAPs lies in the different domains where similarity measures are calculated. Correlation and independence are time-domain measures, which utilize time samples to measure similarities among different spatial locations (voxels in fMRI and nodes in EEG cortical tomography), and, therefore, must be calculated over a time window (typically in tens of seconds to tens of minutes dependent on the factors such as signal type, sampling rate, research question being asked, etc.). On the contrary, the similarity measures used to obtain CAPs are defined in the spatial domain, which are calculated between different temporal locations (individual timeframes). It turns out that CAPs can capture transient events with lifetimes (<100 ms) several orders shorter than the typical time window sizes used for calculating time-domain measures. While the present study and our previous studies (Ding et al., 2022; Shou et al., 2022) demonstrate the CAPs in EEG, similar transient events in fMRI using the concept of CAPs have also been successfully demonstrated (Liu et al., 2013). The present study together with our previous studies (Ding et al., 2022; Shou et al., 2022) further reveal that the use of different distance measures in the same clustering algorithms can lead to detection of different large-scale transient neuronal patterns. In contrast, the uses of different time-domain measures (e.g., correlation vs independence) typically lead to similar spatial constructs of RSNs (Petersen et al., 2000). Multiple neuronal processes also address why there has been a gap of missing spatial similarities between transient EEG CAPs (Shou et al., 2022) and time-averaged EEG RSNs (Yuan et al., 2016) despite high spatial similarities exist between transient fMRI CAPs and fMRI RSNs (Liu & Duyn, 2013). Our present results indicate that such spatial similarities also exist between EEG CAPs and EEG RSNs but have been overlooked mainly due to a methodological parameter, not due to different signal types (EEG vs fMRI).

One of the key assumptions in the k-means algorithm is that each individual timeframe belongs exclusively to one cluster (i.e., one CAP). This temporal exclusivity restricts the ability to model temporally overlapping or concurrent neural processes. Moreover, the averaged spatial patterns of CAPs (both at individual and group levels) only reflect the dominant neuronal co-activation patterns from all timeframes assigned to the same CAP with differences of nonoverlapping components between individual occurrences of that same CAP likely being filtered out during averaging. Furthermore, it is reasonable to assume that some timeframes within the entire resting EEG recordings might only contain noise-like patterns especially considering the transient nature of neuronal events being detected. Their “hard” assignments to individual clusters might add biases on estimating values for temporal measures (e.g., lifetime). Future studies need to consider how to add a cluster for “noise-like” timeframes to address such biases. However, despite temporal constraints of exclusivity, CAPs are not restricted spatially as the same set of brain regions can be involved in multiple CAPs. Actually, several fMRI studies have argued that fMRI CAPs could be considered as the combination or partial decomposition of time-averaged RSNs (Gutierrez-Barragan et al., 2019; Karahanoğlu & Van De Ville, 2015; Mawla et al., 2023). Our EEG CAPs in the present study exhibit similar phenomena of combinations and partial decompositions, for example, in the visual systems (Fig. 2) and in the spatially hierarchical subsets of CAPs revealed by varying k values (Fig. 9). Similar methodological considerations on constraining either spatial or temporal domain also exist in methods for estimating time-averaged RSNs, where enforcing independences to either spatial or temporal dimension of data have led to spatial ICA and temporal ICA (Calhoun et al., 2001). When underlying neuronal activations are not strictly separable in both space and time, these methods yield suboptimal separation, for example, missing the motor RSN on the right hemisphere (Fig. 2). Therefore, it is important to understand that results from different approaches might reflect different aspects of underlying neuronal activations and need to be interpreted accordingly. Such approach differences can also explain some differences observed between CAPs and RSNs in the present study, for example, two bilateral parietal patterns in CAPs but two lateral parietal patterns in RSNs (Fig. 3).

It is also important to note that the use of different distance measures (i.e., L1-norm vs correlation) and different parcel-based data (i.e., anatomical and functional parcels) in the same k-means algorithm leads to the detections of different large-scale transient neuronal activation patterns, which could be explained by varying sensitivities of different model choices to different neuronal activation patterns. For example, the L1-norm distance measures the absolute difference in magnitude that characterizes how far data points are from each other in the high-order space, which leads to the detection of a set of CAPs that mainly distinguished themselves in their global magnitudes (Shou et al., 2022). Meanwhile, the correlation distance measures pattern similarity regardless of magnitude that characterizes how the values of data points in all dimensions of the high-order space change together, which reveals more spatially structured patterns (i.e., RSN-like patterns) in the present study. While both studies use time course data from anatomical parcels, the use of time course data from functional parcels, that is, based on ICs (Ding et al., 2022), seems to have sensitivities to both CAPs of global patterns and spatially structured patterns (see discussions in Section 4.1). In terms of global coactivation patterns, the use of functional parcels only reveals two global CAP patterns (one global negative and one global positive), while the use of anatomical parcels reveals most of CAPs of global patterns with small-to-moderate spatially structured variations on top (Shou et al., 2022). This is consistent with the interpretation of global CAPs that might be caused by propagation (Shou et al., 2022), which make them temporally dependent and, therefore, inseparable using IC-based functional parcels. Another potential factor contributing to such a difference might be the curse of dimensionality, that is, 14 functional parcels (Ding et al., 2022) vs 100 anatomical parcels (Shou et al., 2022), which states that clusters are more challenging to find in large dimension space due to excess of dimensions inflating the distance metric (Aggarwal et al., 2001; Domingos, 2012). While the correlation distance also suffers from the curse of dimensionality, its insensitivity to overall amplitudes possibly allows it to resolve spatially structured CAPs even in higher-dimensional parcellations.

While the present study constrains the investigation of CAPs in the alpha band, the detections of CAPs in broader frequency bands beyond the alpha band are highly expected if the hypothesized link between CAPs and RSNs from the present study is true, as EEG/MEG RSNs of similar spatial patterns have been detected in both broadband signals (Yuan et al., 2016; De Pasquale et al., 2010) and multi-band signals (Liu et al., 2017; Brookes et al., 2011). Furthermore, large-scale oscillations have suggested cross-frequency coordination among frequency-specific components (e.g., alpha-to-gamma (Jensen & Colgin, 2007)) that could be possibly detected as CAPs if EEG/MEG data from multiple corresponding bands could be analyzed simultaneously. If exists, such cross-frequency CAPs might explain why networks of similar functions are operated at different spectral ranges at different anatomic locations (Vidaurre et al., 2018) (see Section 4.1). Considering the transient nature of CAPs in timescales, it is believed to have better sensitivity in detecting such brain-wide events that are potentially less recurring than RSNs that need stable patterns over longer time windows (see Section 4.3). Future studies expanding from the alpha band to wide frequency ranges can address these topics that are expected to provide more sophisticated insights on brain-wide communication mechanisms.

## Supplementary Material

Supplementary Material

## Data Availability

The data used in this study are not publicly available due to data sharing restrictions from the IRB but are available from the corresponding author through a data use agreement upon reasonable request.
